# Platelet‐Rich Plasma for Rhinitis Medicamentosa: A Promising Histopathological Study in an Animal Model

**DOI:** 10.1002/ohn.1303

**Published:** 2025-05-16

**Authors:** Kamil Gokce Tulaci, Salih Yayman, Erhan Arslan, Hasan Canakci, Tugba Tulaci, Gülay Turan, Omer Hizli, Hasmet Yazici

**Affiliations:** ^1^ Departments of Otorhinolaryngology Head and Neck Surgery, Faculty of Medicine Balikesir University Balikesir Turkey; ^2^ Departments of Otorhinolaryngology Head and Neck Surgery Ministry of Health Ankara Etlik City Hospital Ankara Turkey; ^3^ Department of Pathology, Faculty of Medicine Balikesir University Balikesir Turkey

**Keywords:** corticosteroids, oxymetazoline, platelet‐rich plasma, rat model, rhinitis medicamentosa

## Abstract

**Objective:**

To investigate whether platelet‐rich plasma (PRP) has an efficacy on histopathologic changes in rhinitis medicamentosa (RM) in a rat model.

**Study Design:**

Experimental animal study.

**Setting:**

University animal laboratory.

**Methods:**

Forty Wistar albino rats were randomly assigned to five groups to assess the effects of various treatments on RM. Group 1 received intranasal normal saline solution (NSS) for 60 days and served as the negative control. Group 2 was administered intranasal oxymetazoline for 60 consecutive days to induce RM. After decapitation, histopathological evaluation confirmed the development of RM in group 2. The remaining three groups were then similarly treated with oxymetazoline for 60 days. Following this period, group‐specific treatments were applied for an additional 15 days: group 3 received NSS, group 4 was treated with intranasal corticosteroids, and group 5 received intranasal PRP. Nasal mucosal samples were harvested and subjected to histopathological evaluation to determine treatment‐related changes.

**Results:**

Intranasal PRP treatment significantly reduced submucosal edema and improved submucosal gland degeneration (SGD) scores in the nasal mucosa. Moreover, PRP treatment led to a greater reduction in total histopathological score compared to steroid treatment (*P* = .007).

**Conclusion:**

This study demonstrated that PRP administration effectively ameliorates submucosal edema, SGD, and total histopathologic score in experimentally induced RM. Given that PRP is an autologous product with a low risk of side effects, it may serve as a promising alternative to steroids in the treatment of RM.

Rhinitis medicamentosa (RM) is a form of nonallergic rhinitis resulting from the prolonged or excessive use of topical nasal decongestants, with a reported prevalence ranging from 1% to 7%.^1^, ^2^ Histopathologically, features of RM include epithelial degeneration (ED), vascular congestion, ciliary loss, goblet cell hyperplasia, subepithelial edema, and chronic inflammation.^3^

Despite its clinical significance, RM remains difficult to manage due to the absence of standardized treatment protocols and inconsistent patient responses.^1^, ^4^ The current standard of care typically involves the immediate cessation of the offending decongestant, nasal irrigation with 0.9% sodium chloride (normal saline solution [NSS]), and administration of intranasal corticosteroids.^4^, ^5^

Platelet‐rich plasma (PRP) has gained prominence in various medical fields due to its regenerative, anti‐inflammatory, and healing properties, along with its ease of preparation and favorable safety profile. In otorhinolaryngology, PRP has demonstrated therapeutic potential in procedures such as myringoplasty, management of olfactory dysfunction, repair of oroantral fistulas, and mucosal regeneration in atrophic rhinitis.^6^, ^7^, ^8^, ^9^ These beneficial effects are primarily attributed to the abundance of growth factors in PRP, such as transforming growth factor‐beta (TGF‐β), vascular endothelial growth factor (VEGF), and platelet‐derived growth factor (PDGF), which promote tissue repair and modulate inflammation.^10^

To date, no studies have investigated the potential role of PRP in the treatment of RM, a condition characterized by persistent inflammation, edema, and structural damage to the nasal mucosa and turbinates. Addressing this gap, the present study aims to evaluate the histopathological effects of PRP in a rat model of RM.

Although animal models cannot fully replicate the human disease, rat models are widely employed in RM research due to their reproducibility, ability to mirror key histopathological features, and the ethical and practical challenges associated with obtaining human nasal tissue samples.^1^, ^3^, ^11^

In light of these considerations, this study investigates whether PRP exerts therapeutic effects on the histopathological changes associated with RM in a rat model, based on its well‐documented regenerative and anti‐inflammatory properties.

## Materials and Methods

This study was conducted at the Experimental Animals Research and Application Center, Balikesir University, following ethical approval from the Balikesir University Experimental Animal Ethics Committee (approval no. 2022/7‐1).

A total of 40 healthy male Wistar albino rats (weighing 280‐300 g) were included. Animals were housed in groups of eight per metal cage under standardized laboratory conditions (temperature: 21°C; humidity: 40%‐60%; 12‐h light/12‐h dark cycle) with unrestricted access to food and water.

### Preliminary Animal Examination, Grouping, and Drug Administration

All animals underwent a 1‐week acclimatization period before the initiation of experimental procedures, during which no systemic or nasal pathology was observed. Following the initiation of oxymetazoline administration, all rats were observed daily for signs of nasal irritation or abnormal behavior, such as sneezing, nasal scratching, or respiratory difficulty. No remarkable symptoms were observed during this period.

The rats were randomly assigned to five groups (n = 8 per group) as outlined in Table 1.

**Table 1:** Experimental Groups and Treatments

Group	Treatment description
Group 1 (control group)	Normal saline solution (0.9% sodium chloride) for 60 d.
Group 2 (oxymetazoline group)	Oxymetazoline for 60 d.
Group 3 (NSS group)	Oxymetazoline for 60 d, followed by normal saline solution for 15 d.
Group 4 (steroid group)	Oxymetazoline for 60 d, followed by mometasone for 15 d.
Group 5 (PRP group)	Oxymetazoline for 60 d, followed by PRP for 15 d.

Abbreviations: NSS, normal saline solution; PRP, platelet‐rich plasma.

All intranasal drug administrations were performed using a micropipette by the same operator to ensure consistency.

The study consisted of five groups:

- Group 1 (control): Received 50 μL of 0.9% sodium chloride (NSS; Eczacıbaşı) in each nasal passage three times daily for 60 days.
- Group 2 (oxymetazoline): Received 50 μL of 0.025% oxymetazoline (Iliadin; Merck) in each nasal passage three times daily for 60 days.

At the end of 60 days, animals in groups 1 and 2 were sacrificed via decapitation to assess whether RM had developed. Histopathological analysis confirmed RM development in group 2.

Groups 3 to 5 underwent the same oxymetazoline protocol as group 2 for 60 days. Following confirmation of RM in group 2, each group received one of the following treatments intranasally for 15 days:

- Group 3 (NSS): 50 μL of NSS was applied to each nasal passage twice daily.
- Group 4 (steroid): 25 μL of 0.05% mometasone furoate monohydrate (Nazoster; Santa Farma) was administered to each nasal passage twice daily.
- Group 5 (PRP): 50 μL of PRP was administered topically to each nasal passage twice daily intranasally using a micropipette (Table 1).

The 15‐day treatment duration was selected based on previous experimental studies and the expected time frame for PRP‐induced regenerative activity.^3^, ^11^, ^12^, ^13^, ^14^

Mometasone furoate was administered at a volume of 25 μL per nostril twice daily. This dose was selected to provide sufficient contact with the nasal mucosa while minimizing systemic absorption via swallowing, thereby reducing the risk of systemic steroid effects that could potentially confound histopathological outcomes.

### PRP Preparation

PRP was prepared using eight age‐matched donor Wistar rats. Blood (5‐10 mL) was obtained via cardiac puncture, and the donor animals were then euthanized by decapitation. PRP was prepared using the T‐Lab PRP KIT® (T‐Biotechnology Laboratory). Whole blood was collected into tubes containing 3.2% sodium citrate and centrifuged at 2900 rpm for 4 minutes using an angled rotor centrifuge. The PRP layer was aspirated with the kit‐supplied needle, transferred into a resuspension tube, and gently mixed for 30 seconds. The prepared PRP was distributed into 30 individual 1‐mL syringes and stored at −80°C until use.

### Specimen Collection, Preparation, and Examination

Following the treatment period, animals in groups 3 to 5 were sacrificed via decapitation. After removal of the scalp and dissection of the mandible, a coronal section was made approximately 2 mm anterior to the orbital level. Nasal tissues anterior to the incision were excised, fixed in 10% neutral buffered formalin, and randomly coded for blinded histopathological evaluation.

Samples were fixed for 24 hours in formalin and decalcified in nitric acid for 72 hours. Following decalcification, 2‐mm‐thick coronal sections were rinsed under running water for 4 hours, processed using standard protocols (dehydration, clearing, and paraffin embedding), and sectioned into 4‐μm‐thick slices. Sections were stained with hematoxylin and eosin and examined under light microscopy at magnifications of ×40, ×100, ×200, and ×400.

In RM, submucosal edema (SE) and chronic inflammation are considered the key features responsible for nasal obstruction, the primary complaint associated with the condition.^2^, ^3^, ^15^, ^16^, ^17^, ^18^

**Figure 1:**
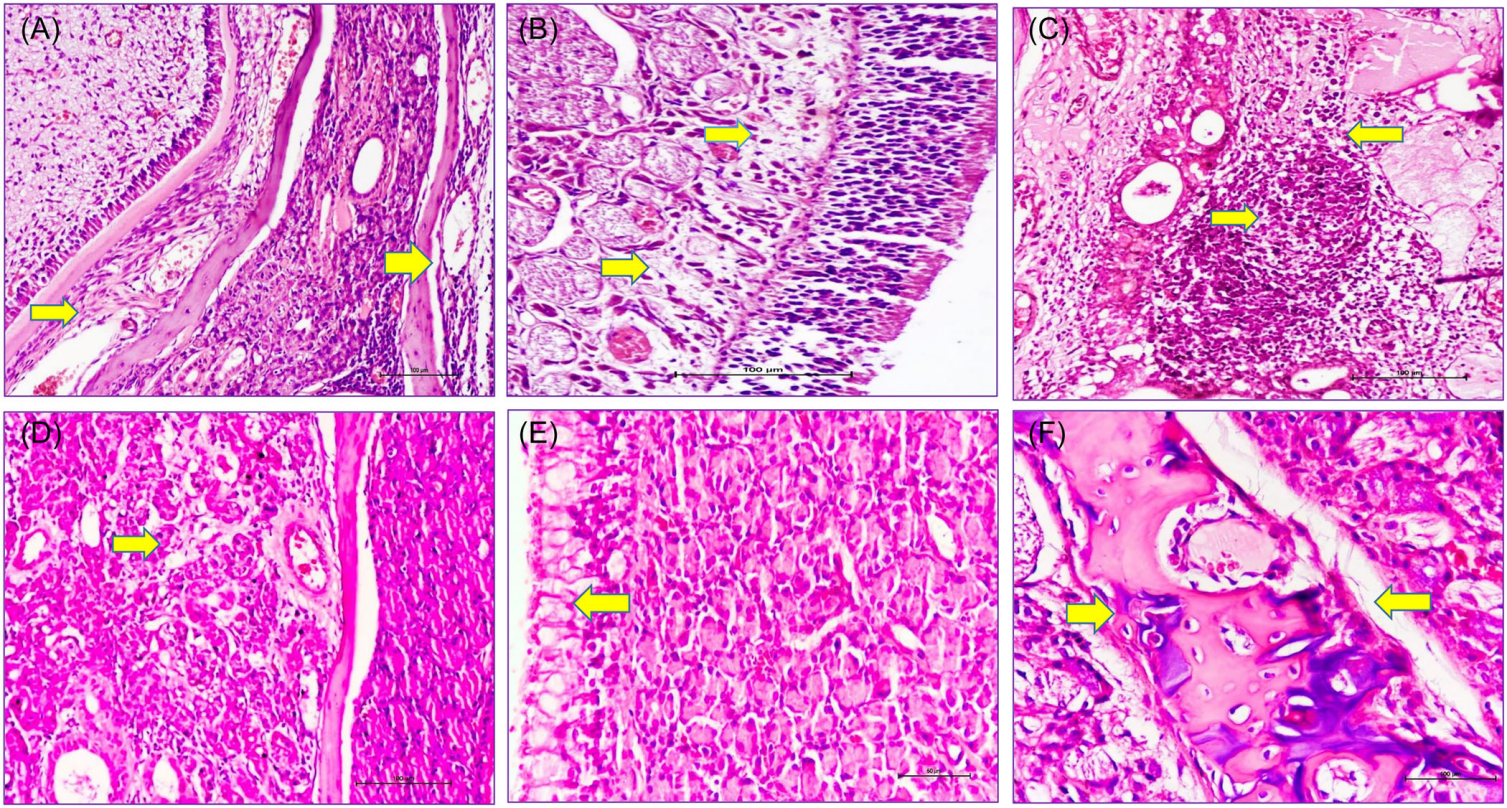
The histologic evaluation of the nasal mucosa. (A) Congestion, dilated vascular structures, score 3, hematoxylin and eosin (x100) (yellow arrow). (B) Edema, interstitial fluid accumulation, score 2, hematoxylin and eosin (x100) (yellow arrow). (C) Inflammation, lymphocytic infiltration in the stroma, score 3, hematoxylin and eosin (x100) (yellow arrow). (D) Submucosal gland degeneration, score 1, hematoxylin and eosin (x100) (yellow arrow). (E) Vacuolar degeneration in epithelial cells, score 2, hematoxylin and eosin (x100) (yellow arrow). (F) Cartilage degeneration, score 2, hematoxylin and eosin (x100) (yellow arrow).

Histopathological assessments were performed to evaluate several key parameters, including ED, basement membrane thickening (BMT), SE, inflammation, congestion, submucosal gland degeneration (SGD), and cartilage surface degeneration (CSD) (Figure 1).

These parameters were assessed semiquantitatively in the areas where histopathological changes were most prominent by an experienced pathologist. Histopathological findings were scored on a scale from 0 to 3: 0 = none, 1 = mild, 2 = moderate, and 3 = severe^3^ (Table 2). The scoring was based on visual assessment of the severity and extent of each finding and therefore reflects a subjective evaluation. However, the pathologist was blinded to the group allocation and was unaware of whether the specimens belonged to the PRP, steroid, or saline‐treated groups, thus minimizing potential bias in the assessment.

**Table 2:** Histopathological Scoring Criteria

Score	Findings
0 none; 1 mild; 2 moderate; 3 severe	Epithelial degeneration (ED)
0 none; 1 mild; 2 moderate; 3 severe	Basement membrane thickening (BMT)
0 none; 1 mild; 2 moderate; 3 severe	Submucosal edema (SE)
0 none; 1 mild; 2 moderate; 3 severe	Separation of the basement membrane
0 none; 1 mild; 2 moderate; 3 severe	Inflammation
0 none; 1 mild; 2 moderate; 3 severe	Congestion
0 none; 1 mild; 2 moderate; 3 severe	Submucosal gland degeneration (SGD)
0 none; 1 mild; 2 moderate; 3 severe	Cartilage surface degeneration (CSD)

### Statistical Analysis

The results of the study were presented as median (min‐max) due to the nonnormal distribution of the data (the normality of the data distribution was assessed using the Shapiro‐Wilk test) and the small sample size in each group (n** =** 8). Accordingly, nonparametric statistical tests were applied. Comparisons between two groups were performed using the Mann‐Whitney *U* test, and comparisons between three groups were performed using the Kruskal‐Wallis test followed by pairwise comparisons with Bonferroni correction. Pairwise comparisons among the NSS, steroid, and PRP groups were performed using Mann‐Whitney *U* tests with Bonferroni‐adjusted *P*‐values. All statistical analyses were conducted using SPSS 25.0 software for Windows (SPSS Inc.). A *P*‐value less than .05 was considered statistically significant.

## Results

A total of 40 Wistar albino rats were included in the study. In the initial phase, the histopathological parameters of the control and oxymetazoline groups were compared to confirm the development of RM. Although the median scores for ED, BMT, and CSD did not significantly differ between the oxymetazoline and control groups (*P* = .105, *P* = 1, and *P* = .442, respectively), the median scores for SE, inflammation, congestion, and SGD were significantly higher in the oxymetazoline group compared with the control group (*P* < .001, *P* = .001, and *P* = .002, respectively). Furthermore, the median total histopathological score was significantly greater in the oxymetazoline group than in the control group (*P* < .001) (Table 3). These findings confirm the successful induction of RM in the experimental model.

**Table 3:** Initial Comparison Results of the Control Group and the Oxymetazoline Group to Confirm RM Development^a^

	Control group	Oxymetazoline group	*P*‐value
Epithelial degeneration	0 (0‐0)	1 (0‐1)	.105
Basement membrane thickening	0 (0‐0)	0 (0‐0)	1
Submucosal edema	0 (0‐0)	1 (1‐1)	**<.001**
Inflammation	0 (0‐1)	1 (1‐3)	**.001**
Congestion	0 (0‐0)	2 (1‐2)	**<.001**
Submucosal gland degeneration	0 (0‐0)	1 (0‐2)	**.002**
Cartilage surface degeneration	0 (0‐0)	0 (0‐1)	.442
Total score	0 (0‐1)	6 (4‐9)	**<.001**

Bold values indicate statistical significance at *p* < 0.05.

Abbreviation: RM, rhinitis medicamentosa.

^a^ Data are presented as median (min‐max).

In the comparison of the NSS, steroid, and PRP treatment groups using Kruskal‐Wallis analysis, no significant differences were observed in the median scores for ED, BMT, inflammation, congestion, or CSD (*P* = .719, *P* = .079, *P* = .072, *P* = .089, and *P* = .222, respectively). However, significant differences were found in the median SE, SGD, and total histopathological scores among the three groups (*P* = .013, *P* = .014, and *P* = .006, respectively; Table 4).

**Table 4:** Comparison of Histopathologic Parameters Among the Study Groups^a^

	RM received NSS	RM received steroid	RM received PRP	*P*‐value^b^
Epithelial degeneration	0 (0‐1)	0 (0‐2)	0 (0‐1)	.719
Basement membrane thickening	0 (0‐0)	0 (0‐1)	0 (0‐0)	.079
Submucosal edema	1 (1‐2)	1 (1‐3)	1 (0‐1)	**.013**
Inflammation	1 (1‐2)	1 (0‐2)	1 (0‐1)	.072
Congestion	1 (1‐2)	2 (1‐3)	1 (1‐2)	.089
Submucosal gland degeneration	2 (1‐2)	1 (1‐2)	1 (0‐1)	**.014**
Cartilage surface degeneration	0 (0‐1)	1 (0‐2)	0 (0‐1)	.222
Total Score	6 (4‐8)	7 (5‐11)	3 (1‐7)	**.006**

Bold values indicate statistical significance at *p* < 0.05.

Abbreviations: NSS, normal saline solution; PRP, platelet‐rich plasma; RM, rhinitis medicamentosa.

^a^ Data are presented as median (min‐max).

^b^ Kruskal‐Wallis test.

Pairwise comparisons among the NSS, steroid, and PRP groups (Table 5) showed that the median SE score in the PRP group was lower than that in the steroid group, though this difference did not reach statistical significance (1 [0‐1] vs 1 [1‐3], *P* = .069). The median SE score did not significantly differ between the NSS and steroid groups (*P* = 1); however, it was significantly lower in the PRP group than the NSS group (*P* = .017), indicating a notable reduction in SE with PRP treatment.

**Table 5:** *P*‐Values of Pairwise Comparisons Among the NSS, Steroid, and PRP Groups

	*P*‐values		
	Submucosal edema	Submucosal gland degeneration	Total score
NSS versus steroid	1	1	1
NSS versus PRP	**0.017**	**0.02**	0.055
Steroid versus PRP	0.069	0.066	**0.007**

Bold values indicate statistical significance at *p* < 0.05.

Abbreviations: NSS, normal saline solution; PRP, platelet‐rich plasma.

Similarly, the median SGD score in the PRP group was lower than that in the steroid group, although the difference approached but did not reach statistical significance (1 [0‐1] vs 1 [1‐2], *P* = .066). A significant difference was observed between the PRP and NSS groups, with lower SGD scores in the PRP group (*P* = .02). No significant difference in SGD was found between the NSS and steroid groups (*P* = 1), suggesting a potential positive effect of PRP on reducing SGD.

Regarding the total histopathological score, pairwise comparisons revealed a significantly lower median score in the PRP group than the steroid group (*P* = .007). The total scores were comparable between the steroid and NSS groups (*P* = 1). These findings suggest that PRP treatment may exert a beneficial effect on the nasal mucosa by improving the overall histopathological profile.

## Discussion

This study evaluated the histopathological effects of PRP in a rat model of RM and, to the best of our knowledge, is the first to explore its therapeutic potential in this context. PRP treatment significantly reduced SE, improved SGD scores, and led to a greater reduction in the total histopathological score compared with steroid treatment. These findings suggest that PRP may represent a promising and innovative treatment option for RM.

Despite the widespread use of topical nasal steroids for their anti‐inflammatory and antiedematous effects, no standardized treatment protocol for RM has been established, and the overall efficacy of nasal glucocorticoids remains debated.^1^, ^4^, ^12^, ^19^, ^20^ Experimental studies by Tas et al^11^ and Elwany and Abdel‐Salaam^13^ demonstrated that topical steroids reduced edema and congestion in RM. However, these studies relied solely on light microscopy without statistical analysis or evaluation of potential side effects. In our study, PRP demonstrated comparable efficacy to steroids in reducing SE and SGD scores and was more effective in lowering the total histopathological score. Given its autologous origin and low risk of adverse effects, PRP may offer a viable alternative to steroid treatment in RM.

Other experimental treatments—such as hyaluronic acid, xylitol, and erdosteine—remain investigational, with no standardized protocols for clinical use.^1^, ^3^, ^21^, ^22^ PRP, however, contains a high concentration of growth factors including PDGF, TGF‐β, VEGF, and epidermal growth factor (EGF), all of which play critical roles in tissue regeneration, wound healing, and inflammation control.^23^ This growth factor profile positions PRP as a strong candidate for the treatment of inflammatory and degenerative nasal conditions.^10^

Our results suggest that PRP improves overall nasal mucosal histopathology by significantly reducing SE, SGD, and total histopathological scores. Although SE scores were significantly lower in the PRP group compared with the NSS group, the difference between the PRP and steroid groups did not reach statistical significance. Given the link between SE and nasal obstruction, PRP may provide greater symptomatic relief than steroids in RM. Similarly, although the PRP group had a lower median SGD score than both the NSS and steroid groups, the difference with the steroid group narrowly missed statistical significance. This borderline result suggests a potential advantage of PRP over steroids, though the relatively small sample size may have limited statistical power. Larger studies are warranted to confirm these findings and clarify their clinical relevance.

Interestingly, the congestion score in the steroid group was relatively higher than in the other groups, though the difference was not statistically significant. This may reflect individual variations in vascular response or localized vasomotor effects not fully suppressed by corticosteroids. Given that the difference was not statistically significant, this observation may not represent a true pathological worsening. Nevertheless, it highlights the complexity of tissue response to intranasal steroids and should be further explored in future studies.

Overall, our findings support the potential of PRP as a more favorable treatment option than steroids for RM, as indicated by significantly lower total histopathological scores. This effect may stem from PRP's ability to promote regeneration of damaged epithelial and subepithelial tissues and to reduce inflammation and edema through growth factors such as TGF‐β, EGF, and PDGF.

Although our methodology was carefully designed to produce reliable results, several limitations must be acknowledged. As this study was conducted in an animal model, the direct applicability of the findings to human RM remains uncertain. Although many histological features observed in rats, such as ED, SE, and SGD, are also present in human RM, the clinical relevance of these parallels is not guaranteed. For example, Knipping et al^24^ noted that ultrastructural changes in a rabbit model of RM did not fully align with those observed in human nasal mucosa, underscoring the inherent limitations of animal models. Nevertheless, rodent models are widely used due to their reproducibility, controlled evaluation of histological and therapeutic effects, and the limited availability of human nasal tissue.

Another limitation of this study was the use of homologous PRP instead of autologous PRP due to the limited blood volume in individual rats and to avoid rat wastage. Although homologous PRP could theoretically trigger immune responses, no adverse effects were observed in this study. To minimize variability, PRP was derived from healthy homogenized donor rats matched for age, sex, weight, and strain and housed under identical conditions. Although this differs from clinical autologous applications, our findings still provide valuable insights into the regenerative effects of PDGFs on nasal mucosa.

Additionally, the absence of rhinometric assessments limited our ability to correlate histological improvements with functional nasal airway outcomes. Future studies incorporating histological and physiological metrics would strengthen the translational value of the findings.

Despite these limitations, our study offers promising insights into the potential of PRP for the treatment of RM. Given its successful topical use in other otolaryngological applications, PRP may be suitable for intranasal administration in the form of drops or gel. However, optimal dosage, frequency, and duration remain undefined. Well‐designed randomized controlled trials are needed to evaluate the clinical efficacy of PRP in human RM, including its impact on histopathological and functional outcomes, and to establish standardized treatment protocols.

## Conclusion

This study demonstrated that PRP administration significantly improved SE, SGD, and the total histopathological score in an experimental rat model of RM. Due to its autologous origin and favorable safety profile, PRP appears to be a viable alternative to corticosteroids for RM treatment. Nevertheless, further clinical research is warranted to confirm these findings in humans and to determine the optimal dosage, delivery method, and standardized preparation protocols for PRP.

## Author Contributions

**Kamil Gokce Tulaci**, conceptualization and methodology, software, validation, formal analysis, investigation, resources, data curation, writing—original draft preparation, writing—review and editing, visualization, supervision, project administration; **Salih Yayman**, software, validation, formal analysis, investigation, resources, data curation; **Erhan Arslan**, conceptualization and methodology, software, validation, formal analysis, investigation, writing—original draft preparation, writing—review and editing, visualization, supervision, project administration; **Hasan Canakci**, writing—original draft preparation, writing—review and editing, visualization, supervision, project administration; **Tugba Tulaci**, conceptualization and methodology, software, validation, formal analysis, investigation, resources, data curation, writing—original draft preparation, writing—review and editing, visualization, supervision, project administration; **Gülay Turan**, conceptualization and methodology, software, writing—review and editing, visualization, supervision, project administration; **Omer Hizli**, conceptualization and methodology, software, validation, formal analysis, investigation, resources, data curation, writing—original draft preparation, writing—review and editing, visualization, supervision, project administration; **Hasmet Yazici**, conceptualization and methodology, writing—original draft preparation, writing—review and editing, visualization, supervision, project administration.

## Disclosures

### Competing interests

The authors declare that they have no conflict of interest.

### Funding source

This study was funded by the Scientific Research Projects (BAP) Unit of Balikesir University (project number: BAP 2021/124).
